# A Masquerading and Unconventional Cause of Dynamic Intestinal Obstruction: Strangulated Obturator Hernia

**DOI:** 10.7759/cureus.2124

**Published:** 2018-01-29

**Authors:** Aneesh Suresh, Sakthivel Chinnakkulam Kandhasamy, Ashok Kumar Sahoo, Anandhi Amaranathan, NR Vishnu Prasad

**Affiliations:** 1 General Surgery, Jawaharlal Institute of Postgraduate Medical Education and Research (JIPMER), Puducherry, India.

**Keywords:** intestinal obstruction, obturator hernia, hernia

## Abstract

Obturator hernia is an extremely rare type of abdominal wall hernia occurring mostly in elderly, thin females. It is characterized by the herniation of intra-abdominal contents through the obturator foramen. Symptoms are often nonspecific, and the patient usually presents with an acute or subacute intestinal obstruction. A high index of suspicion is needed in such females presenting with abdominal distention and positive Howship-Romberg signs. Computed tomography of the abdomen and pelvis are often necessary to arrive at a diagnosis, and immediate surgical intervention is recommended. The high postoperative morbidity and mortality are often attributed to a delay in the diagnosis and in initiating treatment. We present a case of a 65-year-old lady with strangulated obturator hernia who underwent emergent, lower midline laparotomy with resection and anastomosis of the small bowel and purse-string repair of the hernial defect.

## Introduction

Obturator hernia is one of the unusual varieties of hernias, which is more common among elderly females. Obturator hernia occurs through the obturator foramen, containing obturator nerves and vessels. It poses a diagnostic conundrum due to its infrequent incidence and nonspecific presentation, making its clinical diagnosis delayed as well as difficult. Hence, obturator hernia has among the highest morbidity and mortality of all abdominal hernias (13%-40%) [1]. Cubillo first contributed the role of computed tomography (CT) in the diagnosis of obturator hernia and since then, contrast-enhanced computed tomography (CECT) has become the mainstay of early and accurate diagnosis [2]. Once diagnosed, it is imperative to address the condition as early as possible to reduce the morbidity and mortality. Various surgical strategies have been devised for the repair of this condition.

## Case presentation

A 65-year-old lady was admitted in the surgical emergency with complaints of acute onset of right-sided groin pain followed by diffuse abdominal pain and distension over a period of one week. She also had a history of obstipation for the past one week. The onset of obstipation was associated with bilious vomiting for the past five days. She had no other significant medical history in the past. On physical examination, she was poorly built and nourished and had signs of dehydration. Her lower abdomen was tense and distended with diffuse tenderness. There were no peritoneal signs but bowel sounds were exaggerated. The digital rectal and vaginal examinations were normal. A local examination revealed a fullness in the right groin region around the pubic tubercle with tenderness along the medial thigh (Howship-Romberg sign). The pain was exacerbated by the adduction of the hip or medial rotation of the thigh.

**Figure 1:**
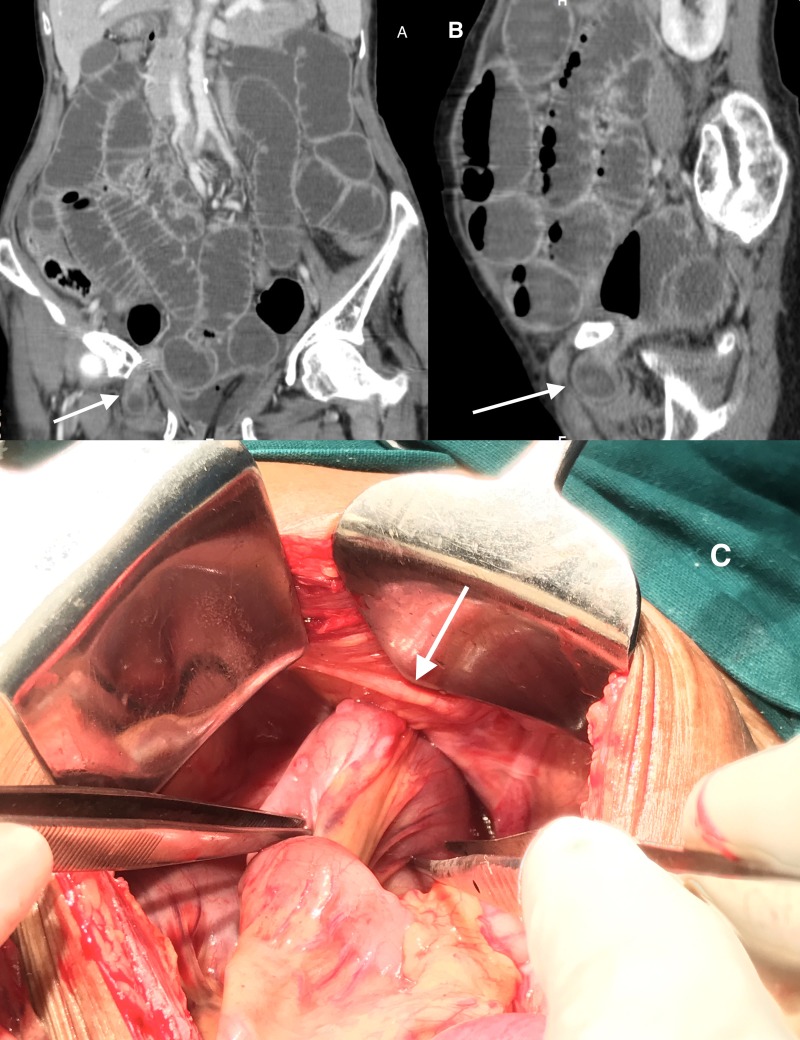
Coronal and sagittal view of CECT image showing small bowel loops entering the right obturator foramen and lying between the pectnieus and obturator externus muscle (arrow). Intraoperative image showing ileal loops entering the right obturator foramen (arrow). CECT: contrast-enhanced computed tomography

Abdominal radiography showed evidence of dilated small bowel loops with multiple air-fluid levels, suggesting intestinal obstructions. An ultrasonography of the abdomen and pelvis showed dilated small bowel loops with decreased peristalsis, but the cause of obstruction could not be identified. Contrast-enhanced computed tomography (CECT) of the abdomen revealed the ileal loop entering through the right obturator foramen and was found between the pectineus and obturator externus (Figure 1A, Figure 1B). A diagnosis of right-sided obstructed obturator hernia was made. The patient was optimized and taken up for emergency laparotomy. Intra-operatively, a 5-cm loop of ileum was found entering the right obturator canal, which was 10 cm proximal to the ileocecal junction (Figure 1C). This loop of bowel was brought into the abdominal cavity. There were pregangrenous changes and a constriction ring in that bowel segment, which was resected, followed by anastomosis. The hernial sac was left in situ and the neck was closed by a nonabsorbable purse-string suture. Her postoperative period was uneventful and she was discharged on postoperative day 10. 

## Discussion

Obturator hernia is nicknamed “little old lady’s hernia” due to its high prevalence in elderly, thin females [1]. It occurs through the obturator canal containing corresponding nerves and vessels. In 1724, Arnaud de Ronsil first described this condition and Henry Ombre, in 1857, credited with its successful repair [1]. It has an incidence of 0.07%-1.0% of all hernias [1]. The male-to-female occurrence of this rare hernia is 6-9:1. Right-sided hernias are more common; the presence of the sigmoid colon covering the left obturator canal is responsible for a lower incidence on that side. Bilateral hernias are seen in 6% of the cases [1,3]. The broader pelvis, with a larger and more horizontal obturator foramen, in older females makes them more susceptible to this condition. Emaciation causes a loss of pre-peritoneal fat and corpus adiposum, which envelop the obturator canal predisposing for obturator hernia.

Acute intestinal obstruction is the most common presentation, which is seen in nearly 90% of the patients, and the mean duration of presentation is approximately four to six days [3]. Obturator hernia, as a cause of small bowel obstruction, is very rare and constitutes only 0.2%-1.6% of cases. Obturator hernia has the highest mortality of all abdominal hernias (13%-40%) [1]. The presentation allies with abdominal pain and distension, vomiting, and constipation/obstipation. The content of the hernial sac in an obturator hernia is usually the small bowel, which is the Richter type in 41%-100% of cases, causing partial or complete intestinal obstruction. Other rare contents include the large bowel, appendix, omentum, bladder, or reproductive organs such as the uterus and ovaries.

The Howship-Romberg sign seen in obturator hernia is because of the compression of the obturator nerve in the obturator canal. This sign is considered to be pathognomonic for obturator hernia but can be elicited in only 15%-50% of the cases. It can be invalidated in the presence of conditions affecting the ipsilateral hip joint, such as osteoarthritis. Hence, the absence of a Howship-Romberg sign does not rule out the diagnosis of obturator hernia. Another sign, the Hannington-Kiff sign, is described as the absence of the thigh adductor reflex in the presence of the patellar reflex. Though the Hannington-Kiff sign is more specific than the previous sign, it is used less frequently. Since the mode of presentation and the signs elicited are usually inadequate for diagnosis, radiological investigations are the cornerstone for diagnosis [3].

The investigations include plain radiographs, contrast studies, herniography, ultrasonography, computed tomography, and magnetic resonance imaging [4]. Computed tomography is considered the most accurate among all the available investigations, but despite increased diagnostic accuracy, it does not reduce the postoperative morbidity and mortality since it is independent of the time lag between the onset of symptoms to presentation [5]. Once diagnosed, urgent surgical exploration is advisable without any further delays, as it might add up to the morbidity.

The surgical approaches include lower midline laparotomy (most common), retroperitoneal, obturator, extraperitoneal, laparoscopic, and a combination of these approaches [6].

The repair of a hernial defect can be done using simple peritoneal repair; otherwise, the interposition of surrounding tissues like aponeurosis, periosteum, muscle, costal cartilage, greater omentum, round ligament, uterine fundus, ovary, bladder wall, or prosthetic material such as a mesh can be used for the repair [6]. Despite surgical repair, the recurrence rate is estimated to be around 10% [6].

## Conclusions

Obturator hernia has the lowest incidence of all abdominal wall hernias. A high index of clinical suspicion is required for diagnosis, especially in old and emaciated females. It poses a diagnostic conundrum due to its rarity and unusual presentation, which makes its diagnosis difficult clinically. Computed tomography is the best imaging modality, which helps in early and accurate preoperative diagnosis. The prompt identification of the condition and urgent surgical exploration is recommended to reduce the morbidity and mortality.

## References

[1] Mantoo SK, Mak K, Tan TJ (2009). Obturator hernia: diagnosis and treatment in the modern era. Singapore Med J.

[2] Cubillo E (1983). Obturator hernia diagnosed by computed tomography. AJR Am J Roentgenol.

[3] Mandarry MT, Zeng S-B, Wei Z-Q, Zhang C, Wang Z-W (2012). Obturator hernia-a condition seldom thought of and hence seldom sought. Int J Colorectal Dis.

[4] Skandalakis LJ, Androulakis J, Colborn GL, Skandalakis JE (2000). Obturator hernia. Embryology, anatomy, and surgical applications. Surg Clin North Am.

[5] Yokoyama Y, Yamaguchi A, Isogai M, Hori A, Kaneoka Y (1999). Thirty-six cases of obturator hernia: does computed tomography contribute to postoperative outcome?. World J Surg.

[6] Rodríguez-Hermosa JI, Codina-Cazador A, Maroto-Genover A, Puig-Alcántara J, Sirvent-Calvera JM, Garsot-Savall E, Roig-García J (2008). Obturator hernia: clinical analysis of 16 cases and algorithm for its diagnosis and treatment. Hernia.

